# Estimating ancestry and heterozygosity of hybrids using molecular markers

**DOI:** 10.1186/1471-2148-12-131

**Published:** 2012-07-31

**Authors:** Benjamin M Fitzpatrick

**Affiliations:** 1 Department of Ecology and Evolutionary Biology, University of Tennessee, Knoxville, TN 37996, USA; 2 National Institute for Mathematical and Biological Synthesis, Knoxville, TN 37996, USA

## Abstract

**Background:**

Hybridization, genetic mixture of distinct populations, gives rise to myriad recombinant genotypes. Characterizing the genomic composition of hybrids is critical for studies of hybrid zone dynamics, inheritance of traits, and consequences of hybridization for evolution and conservation. Hybrid genomes are often summarized either by an estimate of the proportion of alleles coming from each ancestral population or classification into discrete categories like F1, F2, backcross, or merely “hybrid” vs. “pure”. In most cases, it is not realistic to classify individuals into the restricted set of classes produced in the first two generations of admixture. However, the continuous ancestry index misses an important dimension of the genotype. Joint consideration of ancestry together with interclass heterozygosity (proportion of loci with alleles from both ancestral populations) captures all of the information in the discrete classification without the unrealistic assumption that only two generations of admixture have transpired.

**Methods:**

I describe a maximum likelihood method for joint estimation of ancestry and interclass heterozygosity. I present two worked examples illustrating the value of the approach for describing variation among hybrid populations and evaluating the validity of the assumption underlying discrete classification.

**Results:**

Naively classifying natural hybrids into the standard six line cross categories can be misleading, and false classification can be a serious problem for datasets with few molecular markers. My analysis underscores previous work showing that many (50 or more) ancestry informative markers are needed to avoid erroneous classification.

**Conclusion:**

Although classification of hybrids might often be misleading, valuable inferences can be obtained by focusing directly on distributions of ancestry and heterozygosity. Estimating and visualizing the joint distribution of ancestry and interclass heterozygosity is an effective way to compare the genetic structure of hybrid populations and these estimates can be used in classic quantitative genetic methods for assessing additive, dominant, and epistatic genetic effects on hybrid phenotypes and fitness. The methods are implemented in a freely available package “HIest” for the R statistical software ( http://cran.r-project.org/web/packages/HIest/index.html).

## Background

Research on hybrids and hybrid zones offers unique insights into several aspects of evolutionary and ecological genetics [1-6], and natural hybridization might sometimes have a key role in evolutionary diversification and innovation [7-11]. Hybridization can also present a major challenge for conservation when it involves endangered and/or invasive species [12-16]. Therefore, accurate detection and characterization of hybridization is important for both basic and applied biology. Molecular genetic markers are making such analyses accessible across a wide range of organisms, but careful data analysis and interpretation are required to avoid erroneous inferences or misleading communications with non-scientists.

When describing a possible hybrid population, investigators often wish to summarize each individual’s multilocus genotype in a simple and informative way. This usually takes the form of either a hybrid index indicating the proportion of an individual’s ancestors belonging to each “parental” lineage [17-20], or a classification as putative parental, F1, F2, or backcross [21-24]. The hybrid index recognizes that hybrids often form a continuum rather than discrete categories, but the index can be unsatisfactory because it summarizes only one dimension of the genotype. Classification emphasizes the differences between early and later generation hybrids (e.g., F1 and F2 hybrids have the same expected hybrid index $=\frac{1}{2}$ but important differences in the fraction of heterozygous loci). This distinction is important because parental genotypes can potentially be recovered from a population in the early generations of admixture [25], and absence of later generation hybrids might indicate hybrid sterility [26]. However, analyses or management strategies that *assume* discrete classification fail to recognize the continuum of genotypes characteristic of most hybrid zones in the wild, and might perpetuate misleading ideas about the existence of discrete genetic categories [27,28].

Although no summary method is likely to satisfy all needs, the situation can be greatly improved by adding a single calculation so that hybrid genotypes are characterized by estimates of both ancestry (*S*, the axis that arranges all hybrids between two ancestral extremes) and interclass heterozygosity (*H*_*I*_, the axis that distinguishes F1, F2, and recombinant inbred lines). In fact, joint estimates of ancestry and interclass heterozygosity include all of the information in the typical six-type classification because each class has a unique pair of expected values (Table 1) [29-31]. In evolutionary quantitative genetics, early generation hybrid classes are used to study dominance and epistasis precisely because they provide information on *S* and *H*_*I*_, not because the classification itself contains any other information [22,29-32].

**Table 1:** Expected genomic proportions of early generation hybrids

**Class**	***S***	***H***_***I***_	***p***_**11**_	***p***_**12**_	***p***_**22**_
P1	0	0	1	0	0
P2	1	0	0	0	1
F1	1/2	1	0	1	0
F2	1/2	1/2	1/4	1/2	1/4
B1	1/4	1/2	1/2	1/2	0
B2	3/4	1/2	0	1/2	1/2

Each of the genotypic classes generated in the first two generations of admixture has a unique pair of ancestry *S *and interclass heterozygosity ***H***_***I***_[30,31], or equivalently, a unique set of genomic proportions [33], where ***p***_**11 **_is the proportion of the genome homozygous for P1 alleles, ***p***_**22 **_is the porportion homozygous for P2 alleles, and ***p***_**12 **_**= *H***_***I ***_is the proportion of the genome heterozygous for alleles derived from each parental lineage.

Below, I present simple maximum likelihood methods for estimating ancestry and heterozygosity from molecular marker data and explicitly testing the assumption that a discrete classification adequately describes an individual or dataset. I use empirical data and simulations to illustrate these two dimensions of hybridity and assess the reliability of inferences about discrete vs. continuous distributions of hybrid genotypes.

## Methods

### Ancestry and interclass heterozygosity for codominant markers

Buerkle [20] developed a maximum likelihood procedure for estimating the ancestry index *S* from non-diagnostic markers. Here, I generalize his method to jointly estimate *S* and *H*_*I*_(the interclass heterozygosity index) for individual hybrid genotypes given parental allele frequencies. It is useful to express genotypic probabilities using Turelli and Orr’s [33] three genomic proportions: *p*_11 _= proportion of loci with both alleles derived from parental species 1, *p*_22 _= proportion of loci with both alleles derived from parental species 2, and *p*_12 _= proportion with one allele from each species. The system is completely specified by two parameters (because *p*_11_ + *p*_12_ + *p*_22 _= 1), and perfectly represents ancestry and interclass heterozygosity because *H*_*I *_= *p*_12_, and $S=p_{11}+\frac{1}{2}p_{12}$ (Table 1) [32].

The probability of a hybrid being homozygous for allele *j* at locus *i* in terms of the allele frequencies in parental population 1 (*f*_*ij*1_) and population 2 (*f*_*ij*2_), and Turelli and Orr’s [33] genomic proportions is

(1) $$\text{Pr}{\left(j,j\right)}_{i}=p_{11}\;f_{\text{ij}1}^{2}+p_{12}\;f_{\text{ij}1}\;f_{\text{ij}2}+p_{22}\;f_{\text{ij}2}^{2}.$$

And the probability of being heterozygous for alleles *j* and *k* at locus *i*:

(2) $$\;\text{Pr}{\left(j,k\right)}_{i}=p_{11}2\;f_{\text{ij}1}\;f_{\text{ik}1}+p_{12}(f_{\text{ij}1}\;f_{\text{ik}2}+f_{\text{ik}1}\;f_{\text{ij}2})+p_{22}2\;f_{\text{ij}2}\;f_{\text{ik}2}.$$

These probabilities can be generalized to consider any number *A* of ancestral gene pools:

(3) $$\text{Pr}{\left(j,j\right)}_{i}=\overset{A}{\underset{a=1}{\sum }}p_{\text{aa}}\;f_{\text{ija}}^{2}+\overset{A-1}{\underset{a=1}{\sum }}\overset{A}{\underset{b>a}{\sum }}p_{\text{ab}}\;f_{\text{ija}}\;f_{\text{ijb}}.$$

And

(4) $$\;\;\;\;\;\text{Pr}{\left(j,k\right)}_{i}=2\overset{A}{\underset{a=1}{\sum }}p_{\text{aa}}\;f_{\text{ija}}\;f_{\text{ika}}+\overset{A-1}{\underset{a=1}{\sum }}\overset{A}{\underset{b>a}{\sum }}p_{\text{ab}}(f_{\text{ija}}\;f_{\text{ikb}}+f_{\text{ika}}\;f_{\text{ijb}}).$$

These expressions assume alleles were drawn at random from within each parental gene pool when the initial admixture was formed, but do not assume Hardy-Weinberg equilibrium within a hybrid population. Equivalent probability statements were used by Pritchard et al. in developing the Bayesian methods implemented in the program STRUCTURE [19,34,35]. STRUCTURE provides estimates of ancestry that incorporate uncertainty about parental allele frequencies. Using site-by-site analysis [34], it can also give Bayesian estimates of interclass heterozygosity. However the latter method requires mapped markers and has been used only rarely [27,36]. Here, I use likelihood to provide simple estimates of ancestry and heterozygosity that allow analysis of the relationship between these two fundamental dimensions of hybrid genotypes. My estimates depend on given parental allele frequencies (rather than incorporating uncertainty about the ancestral populations) and assume all markers are unlinked or sampled at random with respect to linkage (see below). Despite these limitations, I illustrate the usefulness of considering these two dimensions of hybridity, and hope to encourage further development of methods.

The log-likelihood of a set of genomic proportions for a given hybrid genotype with *n* loci is (following Buerkle [20])

(5) $$\;\ell (p_{11},p_{12},p_{22}|\text{genotype})\;=\;\;\overset{n}{\underset{i=1}{\sum }}\;\left\{\;\;\begin{array}{cc} \text{ln}\text{Pr}{\left(j,k\right)}_{i} & \text{heterozygous}\;\text{loci}\\ \text{ln}\text{Pr}{\left(j,j\right)}_{i} & \text{homozygous}\;\text{loci} \end{array}\right)\;\;.$$

Maximizing this function provides estimates of $Ŝ={\hat{p}}_{11}+\frac{1}{2}{\hat{p}}_{12}$ and ${Ĥ}_{I}={\hat{p}}_{12}$. For diagnostic biallelic markers (*f*_*ij*1 _= 1 and *f*_*ij*2 _= 0), the joint MLE has closed form $Ŝ=x_{11}+\frac{1}{2}x_{12}$ and ${Ĥ}_{I}=x_{12}$, where *x*_11 _is the observed fraction of markers homozygous for species 1 alleles, and *x*_12_ is the observed fraction of markers heterozygous for species 1 and species 2 alleles.

### Dominant Markers

The method can be extended to dominant markers (e.g., AFLP). Assume allele *j* is dominant and *k* is recessive (e.g., for the phenotype of presence/absence of a PCR product at position *i* in a gel). The log-likelihood is

(6) $$\;\begin{array}{l} \ell (p_{11},p_{12},p_{22}|\text{marker}\;\text{phenotype})\\ \;=\overset{n}{\underset{i=1}{\sum }}\left\{\begin{array}{c} \text{ln}\left[\text{Pr}{\left(j,j\right)}_{i}+\text{Pr}{\left(j,k\right)}_{i}\right]\;\text{band}\;\text{present}\\ \ln\text{Pr}{\left(k,k\right)}_{i}\;\text{band}\;\text{absent} \end{array}\right)\;. \end{array}$$

### Implementation

**Figure 1:**
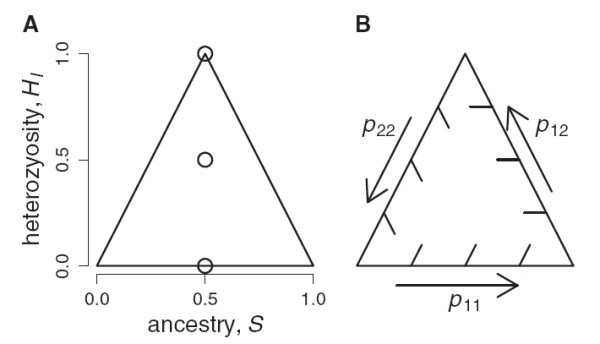
**Sample space of hybrid genomic proportions. **The range of possible hybrid genomic proportions in terms of (A) ancestry and interclass heterozygosity on a bivariate coordinate system, and (B) Turelli and Orr’s [33] genomic proportions on a ternary coordinate system. Labeled circles in (A) show expected *H*_*I *_for three distinct hybrid types, all with *S *= 0.5.

For finding maximum likelihood estimates using equations 5 or 6, I used the general purpose optimization function *optim* in R [37]. The function uses a quasi-Newton optimization algorithm that can handle simple constraints (i.e., proportions must be in the interval [0,1]). However, it sometimes failed for genotypes close to the edge of the triangular sample space (Figure 1), where the likelihood surface is discontinuous. Therefore I implemented two simple Markov Chain Monte Carlo approaches to more thoroughly explore the likelihood surface. The *optim* function can use a built-in simulated annealing (SANN) algorithm, given a function for proposing new estimates. I also wrote a simple MCMC algorithm using Metropolis-Hastings sampling [38]. For both of the these approaches, I wrote a proposal function that draws new genomic proportions (*p*_11_^*″*^*p*_12_^*″*^*p*_22_^*″*^) from a three dimensional Dirichlet distribution centered on the old genomic proportions and with concentration parameter *α*. I.e., the probability density of the proposal distribution is Dir(*αp*_11_,*αp*_12_,*αp*_22_). Larger *α *makes the proposal distribution more concentrated near the current state. For efficiency, starting values were obtained by calculating likelihoods for 100 equally spaced pairs of *S* and *H*_*I*_ on a grid over the sample space and starting the MCMC at the grid point with highest likelihood. For present purposes, I ran the MCMC for 1000 steps (with *α *= 100) and used the pair of estimates with the maximum likelihood as the MLE. The sample space for this problem is simple (Figure 1) and inspection of dozens of likelihood surfaces never suggested the existence of local optima. The quasi-Newton algorithm was unreliable at the edge of the sample space because it could not approximate the local surface as a continuum, not because it was getting stuck at a local optimum.

### Simulations

#### Evolution of ancestry and heterozygosity in admixed populations

To illustrate how the joint distribution of *S* and *H*_*I *_change in the generations following admixture, I created a simple simulation model following Long’s “intermixture” [39]. The simulation begins with individuals from two parental populations in relative frequencies *μ* and 1−*μ*. A first admixed generation of size *N* is formed by randomly drawing *N* pairs of parents with replacement and randomly drawing one gamete from each parent to form *N* diploid offspring. Loci are assumed unlinked, so haploid gametes are formed by randomly drawing one allele from each locus within each parent. This model gives expected frequencies of *μ*^2^, 2 *μ *(1 − *μ*), and (1 − *μ*)^2^ P1, F1, and P2 genotypes in the first generation. Each succeeding generation is formed in the same way by random mating of pairs from the previous generation. I kept track of diploid genotypes to estimate *S* and *H*_*I*_ through time. R code for the simulations is available as Additional File 1.

To illustrate the effect of ongoing gene flow, I repeated the simulations above with stochastic immigration from unchanging parental populations (the continent-island admixture model [40,41]). Each generation, individuals in the hybrid population were replaced by pure parentals with probability *m* (so the expected number of immigrants was *Nm*). Each immigrant was equally likely to be a P1 or P2 genotype.

#### Linkage and sampling of the genome

Linkage among markers is expected to affect the sampling variance (hence reliability) of parameter estimates because linked markers will tend to provide redundant information. The assumption that two markers each provide independent information is violated if they are linked (i.e., if the probability of recombination is less than 0.5). In general this should not be a problem if loci represent a simple random sample with respect to recombinational distance [42]. On the other hand, systematic sampling of a linkage map might provide more reliable estimates if the sample covers most of the genome and the sampling interval does not happen to coincide with some natural periodicity [42], e.g., if the sampled loci were always located near centromeres.

To evaluate the potential effects of linkage on bias and sampling variance, I created a simple linkage model. Each model genome included four diploid chromosomes with 100 loci each. The loci were evenly distributed across two chromosome arms, and one recombination event was modeled per chromosome arm per meiosis (a minimal rate based on mammalian disjunction [43,44]). Recombination breakpoints were drawn with equal probability at any interval on a chromosome arm. This means the recombinational distance between adjacent loci was 2cM. This certainly does not capture all of the complexities of recombination in real genomes [44-46], but it efficiently models a highly structured genome where many randomly sampled markers will be on the same chromosomes.

Using this model, I simulated F2, backcross, and later generation crosses (up to F10) from parental lines with diagnostic alleles at each marker. For comparison, I simulated the same series of cross types allowing free recombination between all markers (400 unlinked markers). For each simulated individual, I recorded the true values of *S* and *H*_*I*_ from all 400 loci, and then estimated *S* and *H*_*I *_from samples of *L* = 3, 10, 20, 30, 40, 50 and 60 loci. For the four-chromosome individuals, I compared estimates using simple random sampling to estimates using systematic sampling where a series of *L* loci at regular 2cM or 10cM intervals was obtained by choosing a single random starting locus. For each simulated individual (1000 of each cross type), I estimated the bias and sampling variance from 1000 random samples of markers for each genomic sample size *L* and sampling regime.

#### Uncertainty of parental allele frequencies

My implementation of the estimators for *S* and *H*_*I *_depends on prior estimates of parental allele frequencies taken as known constants. To briefly illustrate the consequences of inaccurate assumptions about parental allele frequencies, I simulated ten generations of admixture in small populations (*N *= 50) with different sets of actual parental allele frequencies, and then estimated *S* and *H*_*I*_ for each individual under different assumed parental allele frequencies. To evaluate the effect of an overall bias, I used four scenarios: (i) parental populations with *L* diagnostic markers, (ii) *L* diallelic markers with allele frequencies all equal to 0.9 in one lineage and 0.1 in the other, (iii) *L* diallelic markers with allele frequencies all equal to 0.8 in one lineage and 0.2 in the other, and (iv) *L* diallelic markers with allele frequencies all equal to 0.7 in one lineage and 0.3 in the other. For each of these sets of actual parental allele frequencies, I performed estimation under each set of parental allele frequencies as an assumption. I repeated these analyses with *L *= 3 and *L *= 50 to assess how uncertainty interacts with marker number.

To evaluate the effect of balanced inaccuracy, I simulated admixture from parental lineages with 25 diallelic markers with allele frequencies all equal to 0.9 in one lineage and 0.1 in the other, and 25 additional diallelic markers with allele frequencies all equal to 0.7 in one lineage and 0.3 in the other, and then performed estimation assuming all 50 markers had allele frequencies of 0.8 and 0.2. Finally, to assess the impact of having just a few known diagnostic markers, I repeated this analysis replacing one locus of each type with a diagnostic locus, and performed estimation assuming those two were diagnostic but still assuming the other 48 markers had allele frequencies of 0.8 and 0.2.

### Hybrid Classification

**Figure 2:**
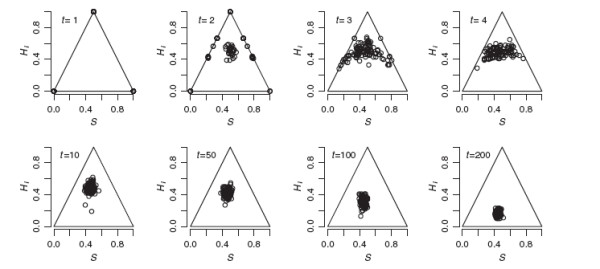
**Evolution of genomic proportions under neutral admixture. **The evolution of genomic proportions under neutral admixture in a simulated population founded by equal numbers from each parental species at t = 0. Population size was held constant at 100 diploids. Genotypes for 100 diagnostic 2-allele codominant markers were tracked over 200 non-overlapping generations of random mating and genetic drift.

Equations (5) and (6) can be used to calculate the likelihood of predefined genotype frequency classes, as in Anderson and Thompson’s program NewHybrids [23]. For example, the likelihood an individual is in the parental 1 genotype frequency class is *ℓ *(*p*_11 _= 1,*p*_12 _= *p*_22 _= 0|marker *phenotype*), the likelihood for the F2 genotype frequency class is *ℓ *(*p*_11 _= 0.25,*p*_12 _= 0.5,*p*_22 _= 0.25|marker *phenotype*), etc. This provides an instructive comparison between the research goals of estimating ancestry and heterozygosity vs. classifying individuals into genealogical categories. First, as noted clearly by Anderson and Thompson [23] among others [21,22], the one-to-one correspondence between genotype frequency class and genealogical class (parental, F1, backcross, etc.) applies only to the first two generations of interbreeding, and arbitrarily similar classes become indistinguishable in practice (Figure 2). Second, for most purposes, the value of knowing the genealogical class is as an indicator of the most likely genotype frequencies, not vice versa [22]. I.e., there is no more genetic information in the classification“backcross to parental 1” than in the set of expected genomic proportions *p*_11 _= 0.5, *p*_12 _= 0.5, *p*_22 _= 0.0 [31]. Finally, the pitfall of classifying samples from a wild population into a limited set of predefined categories is that a best classification will be obtained even if the set of assumed genealogical classes is not relevant (e.g., after more than two generations of admixture).

The most valuable inference from genealogical classification of wild samples is in identifying situations where F1 hybrids are infertile so later generations are never formed [26], or distinguishing brand new hybrid zones from hybrid swarms that are several generations old and therefore unlikely to contain any true parental or F1 individuals [27]. This can be accomplished by evaluating whether any individuals have F1 or parental likelihoods that are (i) sufficiently greater than their likelihoods for other genotype frequency classes to rule those classes out, and (ii) sufficiently similar to the maximum likelihood ancestry and interclass heterozygosity to say the hypothesized classifications cannot be rejected. One approach is to accept a putative classification as credible if the log-likelihood of the best-fit class is over 2 units greater than the log-likelihood of the second best-fit class and within 2 units of the maximum log-likelihood. The first criterion is based on the approximate equivalence of a 2x log-likelihood interval to a 95 percent confidence interval for some distributions [47,48]. The second is based on the conventional penalty of two log-likelihood units for an additional estimated parameter in model selection [49,50]. The classification model can be viewed as having one free parameter (for an individual, once the best-fit class is set to “chosen”, the other five are constrained to “not chosen”), while the continuous model has two (*S* and *H*_*I*_). This approach has the disadvantage of effectively treating the classification as a null model, which is not biologically justified. A better approach is to accept the classification only if its AIC is lower than the AIC of the MLE (in this case, equivalent to a criterion of within 1.0 log-likelihood units of the MLE). Note that the AIC of the best classification cannot be less than the MLE by more than 2 (the case where MLE is identical to the expectation for a class). This approach avoids the pitfall of assuming that individuals fall into a small set of discrete classes, and instead directly evaluates the validity of classification relative to the continuous model MLE.

### Examples

To illustrate inferences based on *S* and *H*_*I*_, I analyzed two published data sets. The first is a sample of hybrid tiger salamanders from a 60-year old hybrid swarm where we expect to find no true parental or F1 individuals [51]. The second is from a hybrid zone between Ensatina salamanders in southern California, where Devitt et al. [52] inferred that a large proportion of individuals in the hybrid zone were in fact F1 hybrids, based on analysis with NewHybrids. To describe ancestry and interclass heterozygosity in these datasets and evaluate support for the existence of true F1 hybrids in the wild, I wrote functions in R [37] to find the joint maximum likelihood estimates of *S* and *H*_*I*_, and to evaluate the likelihoods of the six genotype frequency classes typically of interest (corresponding to the expectations for pure parentals, F1’s, F2’s and first backcrosses in each direction). These functions and others used in this paper are available as a CRAN package called “HIest” (for “hybrid index estimation”) at http://cran.r-project.org/web/packages/HIest/index.html.

#### Introduced x native hybrid swarm in tiger salamanders

Barred Tiger Salamanders (*Ambystoma tigrinum mavortium*) were deliberately introduced from Texas to California in the 1940’s and 1950’s [53]. They have been interbreeding with the native California Tiger Salamander (*A. californiense*) in ponds throughout the Salinas Valley for roughly 20-30 generations. Thus, unless there has been an unknown source of new “pure” Barred Tiger Salamanders in the recent past, it is extremely unlikely that any true F1, F2, or backcross individuals exist in the wild.

Fitzpatrick et al. [51] used 65 putatively diagnostic markers (one allele assumed fixed in each ancestral population) to genotype 255 salamander larvae from five breeding ponds. This example is instructive because diagnostic markers allow use of the closed-form MLE’s as benchmarks for testing the optimization, and the large number of markers gives high precision in evaluating how the distribution of hybrid genotypes varies across populations and whether any populations might contain putatively pure parentals or F1’s.

#### A natural hybrid zone in *Ensatina*

*Ensatina eschscholtzii* is a classic example of the “ringspecies” pattern illustrating the gradual evolution of reproductive isolation and distinctiveness between species taxa [54-58]. Devitt et al. [52] analyzed a narrow hybrid zone in southern California between the distinctive forms *E. e. eschscholtzii* and *E. e. klauberi* using one mitochondrial and three nuclear loci assayed for 335 salamanders densely sampled from across the contact zone. They used NewHybrids [23] and STRUCTURE [19,35] to estimate ancestry (the Baysian *Q*-value estimates the same underlying quantity as *S* here), and classified as “hybrids” the 46 individuals with point estimates between 0.1 and 0.9. Of these, 22 were classified as F1 hybrids and 24 as F2 or backcrosses based on posterior probabilities from NewHybrids. I used their nuclear data (published as online supplementary material) to compare their inferences to my joint likelihood estimation of *S* and *H*_*I*_. This example is instructive because the small number of non-diagnostic markers should give considerably less precision than the tiger salamander example, and because the high frequency of F1 hybrids is biologically significant if the inference is credible.

The nuclear markers used by Devitt et al. [52] were not diagnostic, so I repeated their analysis using the admixture model in STRUCTURE (version 2.3.2) with standard settings to estimate “ancestral” allele frequencies to use as givens (*f*_*ij*1_,*f*_*ij*2_) for my likelihood calculations. I also saved the *Q*-values estimated by STRUCTURE to compare to my MLE’s of *S* (though the inferences are obviously not independent because both depend on the parental allele frequencies inferred by STRUCTURE). This reliance on external estimates of parental allele frequencies is a weakness of my implementation, but I suspect that my approach could be integrated in a fully Bayesian analysis using NewHybrids [23], STRUCTURE [19,34,35], or Introgress [59] as a starting point. To evaluate support for classification of *Ensatina* hybrids into the six standard classes, I once again used both criteria; (i) classification required a difference of two log-likelihood units between the best fit class and any other, and (ii) the best fit class had to have lower AIC than the joint MLE’s of *S* and *H*_*I*_.

### Sampling and false classification

**Figure 3:**
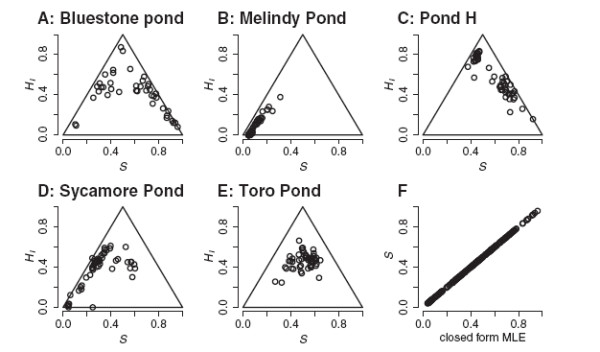
**Distributions of ancestry and heterozygosity in hybrid tiger salamander populations. **Joint maximum likelihood estimates of ancestry and interclass heterozygosity show variation among populations within the California tiger salamander hybrid swarm (A-E). Here, *S *is the proportion of alleles derived from the introduced Barred Tiger Salamander. (F) Illustrates that the joint maximization converges on the closed-form MLE of the ancestry index for diagnostic markers.

To further explore how the number of markers assayed affects erroneous classification, I took the tiger salamander data from Bluestone Pond and Toro Pond (Figure 3a and e) and randomly subsampled markers and recalculated the likelihoods of the six hybrid classes and the joint MLE of *S* and *H*_*I*_. I randomly subsampled three markers (without replacement) and repeated the analysis 1000 times. Then I did the same for samples from 5 to 60 (out of the total of 65) in increments of 5. Given the history of the tiger salamander hybrid swarm and the low frequency of classification using the full dataset, I considered any “successful” classification a false positive.

Because the primary value of classification is in the identification of true F1 or pure parental genotypes [25], I also specifically assessed the frequency with which individuals were classified as parental or F1. For diagnostic markers, this can happen only if an individual is heterozygous at all markers, or homozygous at all markers, respectively. In these cases, the likelihood of the classification is equal to the maximum likelihood, and the AIC-based test will always favor the classification over the continuous model because of the difference in degrees of freedom. However, for small numbers of markers, spurious inference can be made because all markers might be heterozygous or homozygous by chance. For example, in a true F2 or backcross, 50% of markers are expected to be heterozygous and the probability of sampling three heterozygous markers by chance is (1/2)^3 ^= 0.125. To avoid spurious inference, investigators should avoid classifying individuals based on small numbers of markers [21]. For example, the expected fraction of *n* F2’s with all heterozygous genotypes at *L* markers is *α *= *n *(1/2)^*L*^. So, in order to maintain an experiment-wise error rate of *α*, one would need at least

(7) $$L=\frac{\text{log}n-\text{log}\alpha }{\text{log}2}$$

markers. Although this applies precisely only in the case of F2 hybrids and diagnostic markers, it might be taken as a rule of thumb in the absence of other criteria. In the case of the Ensatina data with 46 putative hybrids and three markers, we might expect 5.75 false F1’s and would have wanted 10 markers to keep the error rate near 5%.

## Results and Discussion

### Evolution of ancestry and heterozygosity in admixed populations

Figure 2 shows *S* and *H*_*I*_ from a single random simulation for *N *= 100 with 100 diagnostic codominant markers. The case is typical in showing clear genotypic clusters corresponding to parentals, F1’s, F2’s, and backcrosses in the first two generations, followed by a few generations with high variance of *S*, effectively looking like a continuum between backcross-like and F2-like genotypes (0.25 <*S *< 0.75, *H*_*I*_ near 0.5). By *N*/10 generations almost all individuals are clustered around *S *= *H*_*I *_= 0.5, and the population slowly becomes more homozygous as alleles are lost by drift (*S* remains roughly constant while *H*_*I*_ declines toward zero).

**Figure 4:**
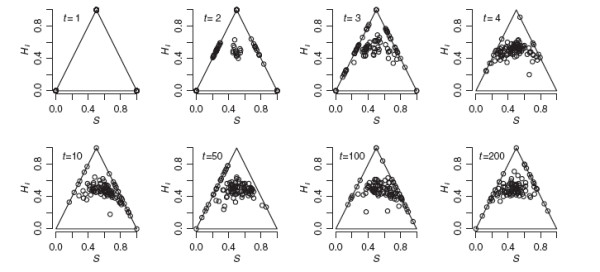
**Evolution of genomic proportions under neutral admixture and immigration.** The evolution of genomic proportions under neutral admixture with ongoing gene flow. The simulated population as founded by equal numbers from each parental species at t = 0. Population size was held constant at 100 diploids. Each generation, resident adults were replaced by pure parental genotypes with probability 0.10 (average gene flow was *Nm *= 10 each generation. Offspring genotypes (before dispersal) for 100 diagnostic 2-allele codominant markers were tracked over 200 non-overlapping generations of immigration, random mating, and genetic drift.

Figure 4 illustrates the effect of ongoing immigration from parental gene pools. With *N *= 100 and *m *= 0.10, a stationary distribution was reached at generation 3. The distribution fluctuates from generation to generation, but a wide range is consistently observed. With lower immigration (*Nm *≤ 1), results were similar to the no-gene-flow scenario in Figure 2, but *H*_*I*_ remained moderate instead of dropping toward zero. With *Nm *= 1, the population settled in a steady state similar to *t *= 50 or *t *= 100 in Figure 2.

**Figure 5:**
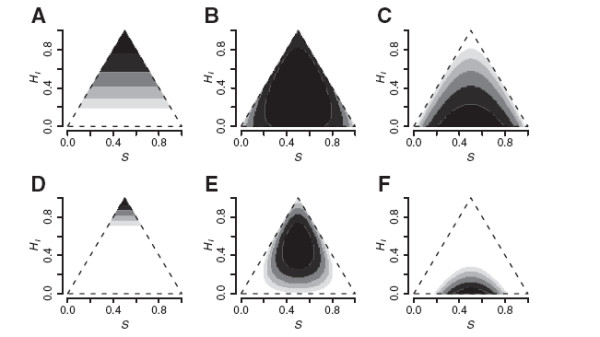
**Likelihood surfaces for codominant markers. **Likelihood surfaces of ancestry (*S*) and interclass heterozygosity (*H*_*I*_) for 10 (A-C) and 40 (D-F) codominant biallelic loci with parental allele frequencies of 0.9 and 0.1. (A) and (D) are F1 hybrids with *S *= 0.5 and *H*_*I *_= 1.0; (B) and (E) are F2 hybrids (*S *= 0.5, *H*_*I *_= 0.5); (C) and (F) are homozygous recombinants (*S *= 0.5, *H*_*I *_= 0.0). Each level of shading covers two units of log-likelihood, so black is within 2 log-likelihood units of the maximum.

**Figure 6:**
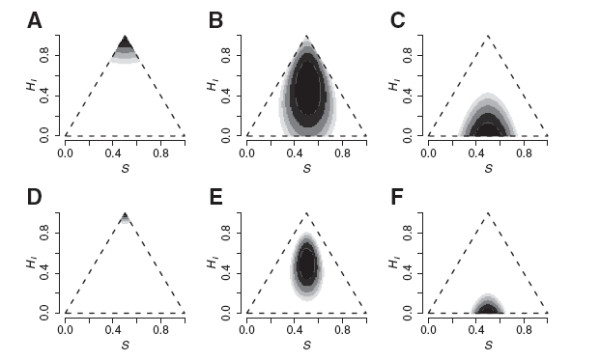
**Likelihood surfaces for dominant markers. **Likelihood surfaces of ancestry (*S*) and interclass heterozygosity (*H*_*I*_) for 100 (A-C) and 300 (D-F) dominant markers for the same three hybrid genotypes as in Figure 5. Dominant allele frequences in the parental species were set to 0.9 for half of the markers and 0.1 for the other half in species 1 and vice versa for species 2. The F1 (A and D) had the dominant phenotype for all markers, the F2 (B and E) was homozygous recessive at $\frac{1}{4}$ of the markers, and the Fn (D and F) was homozygous recessive at $\frac{1}{2}$ of the markers. Each level of shading covers two units of log-likelihood, so black is within 2 log-likelihood units of the maximum.

The same basic patterns can be seen when the loci are not entirely diagnostic (e.g., parental allele frequencies of 0.9 vs 0.1). However, when estimates were based on fewer markers, or less informative markers, it was often impossible to discern discrete genotype clusters by generation 2 (e.g., see Figures 5 and 6).

### Codominant markers

Maximum likelihood estimates of *S* and *H*_*I *_appear consistent and unbiased for known codominant genotypes (Figure 5). Precision depends on the number of markers and how ancestry-informative they are (how different the known parental allele frequencies are). The simplicity of the triangular sample space makes it easy to visualize the likelihood surface for any individual and get a feel for the uncertainty around an estimate. Figure 5 illustrates that a large number of highly informative markers are needed for precise inference about any single genotype.

### Dominant markers

Maximizing the log-likelihood for dominant markers also gives unbiased estimates of *S* and *H*_*I *_(Figure 6). With the inherently lower information content of dominant markers, more markers are needed for precision, as seen in other methodological studies [24,60,61]. These markers are less informative about heterozygosity, hence the oval ellipses in Figure 6. The method works well as long there is a mixture of loci for which the dominant allele is more common in ancestral species 1 and other loci for which the dominant allele is more common in ancestral species 2. The validity of the estimates depend on the validity of homozygous recessive genotypes as information about *p*_11_ and *p*_22_. If, for example, the absence of PCR product or particular band on a gel cannot be interpreted as a homozygous recessive genotype, the marker system should not be used for this or any other method relying on typical population genetic assumptions.

### Linkage and sampling of the genome

Markers sampled at random from a structured genome were indistinguishable from truly unlinked markers in terms of bias and sampling variance of *Ŝ* and ${Ĥ}_{I}$ (Additional File 2: Figures S1-S4). Average bias was indistinguishable from zero for all sampling regimes (Additional file 2: Figures S1, S2), and sampling variance decreased with larger numbers of markers, as expected (Additional file 2: Figures S3, S4). Systematically sampling linked markers affected sampling variance in a manner consistent with statistical intuition [42]. Estimates based on small numbers of tightly linked markers had high sampling variance (i.e., a different sample of markers was likely to give substantially different estimates). However, when coverage of the genome was very good, systematic sampling resulted in lower sampling variance (Additional file 2: Figures S3, S4). For example, given the modeled genome structure (four 200cM chromosomes) 60 markers at 10cM intervals spans 75% of the genome and leads to more reliable estimates of *S* and *H*_*I*_ than a simple random sample of 60 markers. Thus, for systematically sampled genomes with good coverage, support intervals based on my likelihood calculations will be somewhat conservative.

### Uncertainty of parental allele frequencies

Effects of systematic over- or under-estimating differentiation between parental lineages predictably biased hybrid index estimates toward intermediate or extreme values respectively (Additional file 2: Figures S5-S8, Tables S1-S4). For example, if markers are assumed to be diagnostic but actually have frequencies of 0.8 in a parental population, then we would estimate that most pure parental individuals have ancestry Ŝ=0.8 and are heterozygous for foreign alleles with probability $\hat{H_{I}}=2(0.8)(0.2)=0.32$. In contrast, if allele frequencies are assumed to be more intermediate than they truly are in parental lineages (e.g., if parental allele frequencies are estimated from introgressed populations), then estimates will tend to be more extreme than the true values. This situation might result in population samples appearing to have excess F1 hybrids (high *H*_*I*_) and/or parental-like genotypes (high or low *S*).

When an equal number of parental allele frequencies were over- and under-estimated, estimates of *S* were very accurate, but estimates of *H*_*I *_had increased variance and were slightly biased toward extreme values (Additional file 2: Figure S9, Table S5). Adding two known diagnostic loci to the set made negligible difference. Presumably a Bayesian method that could account for uncertainty in parental allele frequencies would ameliorate the slight bias in ${Ĥ}_{I}$ and take better advantage of markers where the quality of information is better. Nevertheless, the simple likelihood approach used here is pretty robust to small errors in the assumed parental allele frequencies, especially if the errors are unbiased.

### Examples

#### Introduced x native hybrid swarm in tiger salamanders

The distributions of individual estimates of ancestry and interclass heterozygosity from the tiger salamander data are illustrated in Figure 4. Populations vary considerably in their joint distributions of *S* and *H*_*I*_. The patterns for Bluestone, Pond H, and Sycamore are consistent with gene flow between populations differing in allele frequencies (Figure 4). Melindy is surrounded by predominantly native populations. Toro is relatively isolated and resembles the simulations of neutral admixture with little immigration (Figure 2). For all except Toro, there seems to be a high concentration of estimates near the maximum possible *H*_*I*_ given *S* (the legs of the triangle), which is consistent with the earlier observation of hybrid vigor in this system [14]. For these diagnostic markers, MLE’s found via MCMC agreed perfectly with the closed form MLE’s (Figure 4f).

**Figure 7:**
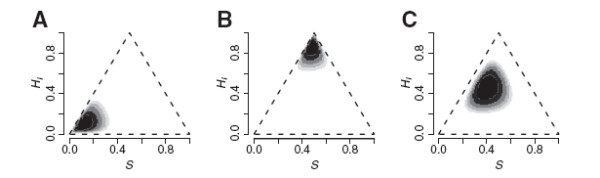
**Example likelihood surfaces for individual tiger salamander hybrids.** Joint maximum likelihood surfaces for three hybrid tiger salamanders from Bluestone Pond (Figure 3A). (A) and (B) are the individuals with the lowest and highest estimated interclass heterozygosity, respectively. (C) is a random draw from the few individuals classified as “F2-like” because the MLE is consistent with *S *= *H*_*I *_= 0.5. Each level of shading represents two log-likelihood units.

Only a small fraction of the sampled tiger salamanders would be classified into one of the six standard genotype frequency classes using the stringent criteria of (i) the best fit of the six had to differ from the others by at least two log-likelihood units, and (ii) the best fit of the six had to have lower AIC than the continuous model MLE. By these criteria, 21 of the 255 larvae would be classified as F2-like (*p*_11 _= 0.25, *p*_12 _= 0.5, *p*_22 _= 0.25) and one as like a backcross to California Salamander (*p*_11 _= 0.5, *p*_12 _= 0.5, *p*_22 _= 0.0). As expected, no larvae would be classified as F1 hybrids or “pure” parental genotypes. In this case, the low level of classification is entirely due to criterion (ii); in 233 of 255 cases, the MLE was a significantly better fit than the best fit of the six classes. Three examples of the very sharply peaked likelihood surfaces typical for this dataset are illustrated in Figure 7.

Thus, with sufficiently high-resolution data, this kind of analysis can show that admixture has been ongoing for more than two generations and the simple hybrid classification scheme of F1, F2, and backcross is clearly inadequate to describe the distribution of genotypes in the wild. Even for Toro Pond, where 14/52 would be classified as F2, the joint distribution of *S* and *H*_*I *_is inconsistent with two generations of admixture because random mating is expected to produce the full array of parental, F1, F2, and backcross genotypes in a population (Figure 2).

#### A natural hybrid zone in *Ensatina*

**Figure 8:**
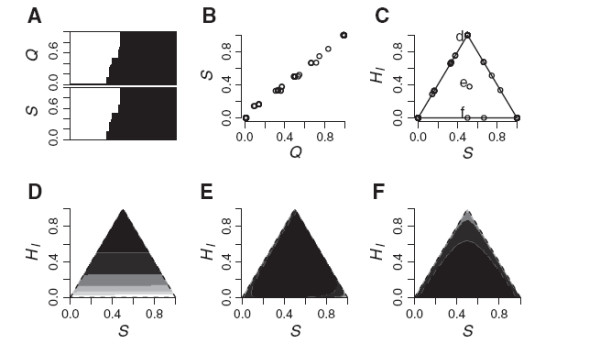
**Estimates of ancestry and heterozygosity in an *Ensatina *hybrid zone. **Joint maximum likelihood estimates of *S *and *H*_*I *_largely corroborate the inferences of Devitt et al. [52] for the *Ensatina *hybrid zone. The MLE ancestry index *S *agrees with the Bayesian *Q*-value from STRUCTURE (A and B). Here *S *is the proportion of alleles derived from *E. e. klauberi*. (C) The distribution of individual estimates is concentrated at *S*= 0 and *S *= 1 (putatively un-admixed individuals). The points labelled “d”, “e”, and “f” correspond to the likelihood surfaces illustrated in (D), (E), and (F). There are 20 coincident points at the top vertex (“d”), 17 of which were “confidently” assigned as F1-like. The point estimate for (e) is almost perfect for an F2-like genotype class (*S *= *H*_*I *_= 0.5), but it cannot be statistically distinguished from a *klauberi* backcross. Simple classification was rejected for (F) because the continuous model MLE had the lowest AIC.

My analysis corroborates the inference that the distribution of genotypes in the *Ensatina* hybrid zone is unusual, but cautions against making strong inferences about hybrid classes based on so few markers. My MLE estimates of the ancestry index *S* are virtually identical to the *Q*-values estimated by STRUCTURE (Figure 8a,b); this is not surprising, as both are based on the clusters inferred by STRUCTURE. Using the same strict criteria as above, my likelihood analysis would classify 112 of their 115 putative *E. e. eschscholtzii* as such and 172 of their 174 putative *E. e. klauberi* as such. My criteria would support F1-like classification for 17 of their 22 putative F1 hybrids. However, even these classifications should be viewed with suspicion in light of the small number of loci used. The remaining 34 salamanders could not be classified as any of the six standard classes. In two cases this was because the MLE was superior to the best classification, but the other 32 genotypes were consistent with more than one class. Because of the uncertainty in the data from these individuals, we cannot confidently accept nor reject the validity of the 2-generation classification vs. later generation hybrids.

The likelihood surfaces fitted to the *Ensatina* data are rather flat (Figure 8d-f). All but three of the 46 putative “hybrids” had maximum likelihood estimates of interclass heterozygosity at the maximum possible value given their MLE values of *S* (Figure 8c). Intuitively, this distribution of genotypes seems consistent with a narrow hybrid zone structured by ongoing immigration of homozygous *E. e. eschscholtzii* and *E. e. klauberi* genotypes (corroborated by other analyses in [52] and [62]). Even so, the extreme concentration of estimates at the edges of the sample space might not hold up with the inclusion of more than three markers (see below), or if there is substantial inaccuracy in the estimates of parental allele frequencies. For example the effective sample size for either parental lineage might be small or many generations of introgression might have made the contemporary populations more similar than the true ancestral lineages. It is important to note that the key conclusions about differential introgression of mtDNA across a narrow hybrid zone [52] are not affected by the validity of the hybrid classification in this case.

### Sampling and false classification

**Figure 9:**
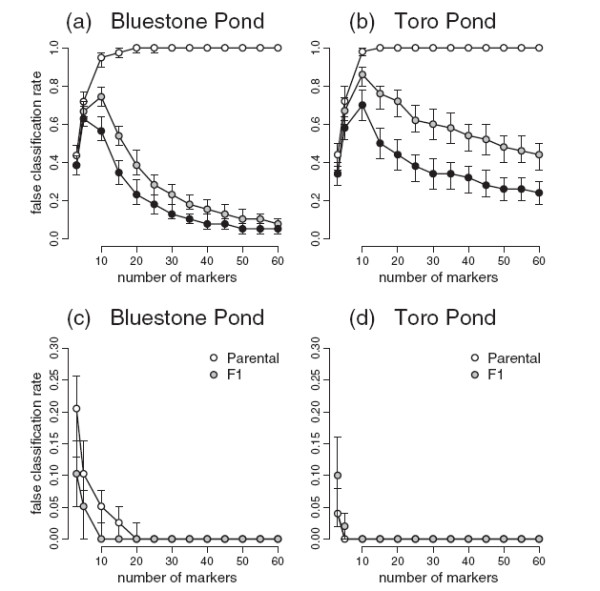
**False classification rates. **False classification rate for subsamples of markers for the Bluestone Pond (a) and Toro Pond (b) tiger salamander data peaked at 10 markers when the typical six category system (limited to parental, F1, F2, and backcross genotypes) could be rejected by the MLE of *S *and *H*_*I*_. Shaded symbols show results for classification based on 2 log-likelihood units; black symbols show results for the AIC criterion. However, with *a priori *limitation to the six categories (open symbols), large numbers of markers invariably lead to a single confident classification for all individuals in the dataset. Points illustrate medians and bars the 0.25 to 0.75 interquartile range (covering 50% of the subsampled data sets for each number of markers). False classification rates specifically for F1 and parental categories (c and d) decline with the number of markers.

When the continuous MLE was compared against classification (using the 2x log-likelihood or AIC criteria), false classification was most common when about 10 markers were subsampled from the tiger salamander data. False classification dropped off for smaller numbers of markers because there was low power to discriminate alternative classes, and dropped off at larger numbers because the increased resolution allowed all six of the classes to be rejected in favor of the MLE (Figure 9). As expected, if only the first criterion was used (i.e., we assume the six standard classes comprise an exhaustive set of possibilities and ignore the MLE), the false classification rate increased monotonically as the number of markers made it easier to reject all five alternatives in favor of the single best-fit class (Figure 9). These analyses show how an investigator’s prior belief in the six category system can affect inference. This study adds yet another cautionary note that it takes rather large numbers of ancestry-informative markers to ensure against false inferences about discrete hybrid classification [21,22,24].

False classification in subsamples of the tiger salamander data was largely attributed to the difficulty of distinguishing F2 and backcross categories from later generation hybrids. Misclassification of later generation hybrids from these populations as parental or F1 was a problem only for small numbers of markers (Figure 9c and d). In Bluestone, with its more dispersed distribution of *S* and *H*_*I *_(Figure 3), a substantial fraction of hybrids could be mistaken for parentals when 10 or fewer markers were used. The tighter distribution of genotypes in Toro Pond made this less of a problem, but a fraction of the Toro Pond animals were consistently classified as F2. Both ponds showed means of ca. 10% F1 misclassification when three markers were used, slightly below the 12.5% that would be expected for a population of F2’s or backcrosses, and substantially below the 37% putative F1’s in the *Ensatina* dataset.

## Conclusions

Hybrids are generally conceived as the genetically mixed descendants of two or more distinct ancestral populations [63]. The mixed genomes of hybrids can be characterized in terms of ancestry (*S*, the fraction of alleles derived from each ancestral group), and interclass heterozygosity (*H*_*I*_, the fraction of loci heterozygous for alleles from each ancestral group). Heretofore, interclass heterozygosity has been used only rarely in analyses of hybridization in the wild, but to great effect [14,27,36,64]. I present an effective method for jointly estimating *S* and *H*_*I*_. The joint likelihood is efficiently expressed in terms of Turelli and Orr’s [33] genomic proportions given information on ancestral allele frequencies. A future improvement would be to jointly estimate ancestral allele frequencies along with individual ancestries and heterozygosities for a sample. This might be achieved in a Bayesian MCMC framework [19,41].

Joint consideration of *S* and *H*_*I *_provides considerably more biological insight than a single ancestry index or classification of hybrids into the limited categories generated in the first two generations of admixture [14,29,32]. My analysis illustrates how reliance on the simple classification scheme (parental, F1, F2, backcross) can be misleading. Classification is appropriate only for study systems in the first two generations of admixture. Even with modest numbers of markers, false acceptance of discrete hybrid classes is likely. More stringent criteria for accepting a classification might be used, but in all cases investigators should carefully consider whether classification of individuals into discrete categories is both realistic and of interest given their research questions. With large numbers of markers (such as the tiger salamander example), the validity of discrete classification can be evaluated and rejected for populations with over two generations of admixture. This might be of biological interest in some cases. In other cases, investigators might be more interested in the MLEs of *S* and *H*_*I*_ than in the likelihood that an individual is truly an F2 hybrid [22].

## Competing interests

The author declares no competing interests, financial or otherwise, regarding this manuscript.

## Supplementary Material

Additional file 1 **Simulations with gene flow. **R code for simulating neutral admixture. Click here for file

Additional file 2 **Supplementary figures and tables.** Figures and tables illustrating effects of linkage and inaccuracy of parental allele frequencies on bias and sampling variance of estimates of *S *and *H*_*I*_. Click here for file
