# Supplementary material for: Estimating ancestry and heterozygosity of hybrids using molecular markers
Source: BMC Evol Biol. 2012 Jul 31;12:131. doi: 10.1186/1471-2148-12-131 (PMC3572440; doi:10.1186/1471-2148-12-131)
Supplement: Additional file 2 — Supplementary figures and tables. Figures and tables illustrating effects of linkage and inaccuracy of parental allele frequencies on bias and sampling variance of estimates of S and HI. [file 1471-2148-12-131-S2.PDF]

Figure S1 – Effects of linkage on bias of ancestry estimates

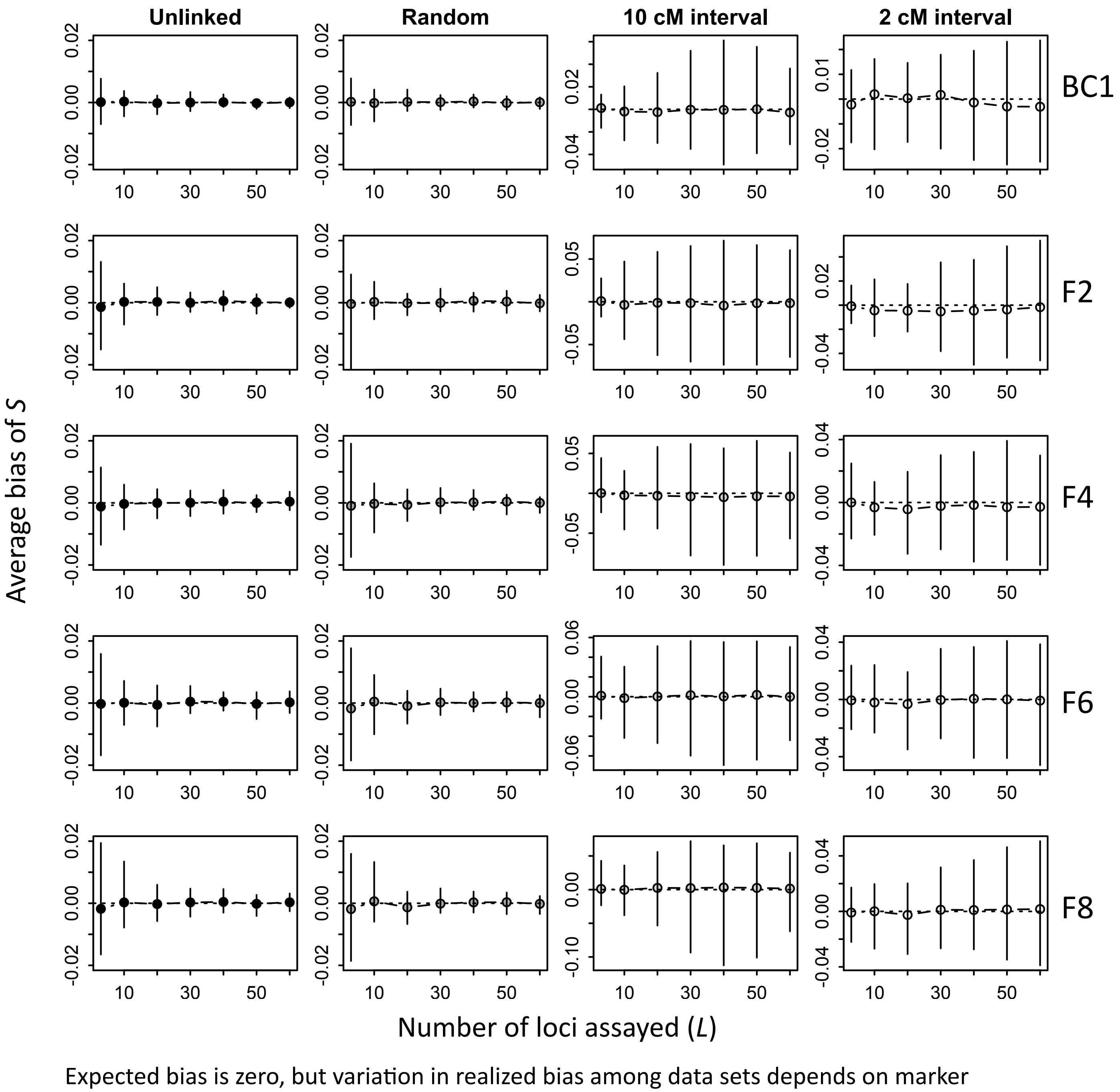

Expected bias is zero, but variation in realized bias among data sets depends on marker number and sampling regime. Means and 95% interquantile ranges (vertical bars) are illustrated for 1000 simulated individuals of each hybrid generation (right margin).

Figure S2 – Effects of linkage on bias of heterozygosity estimates

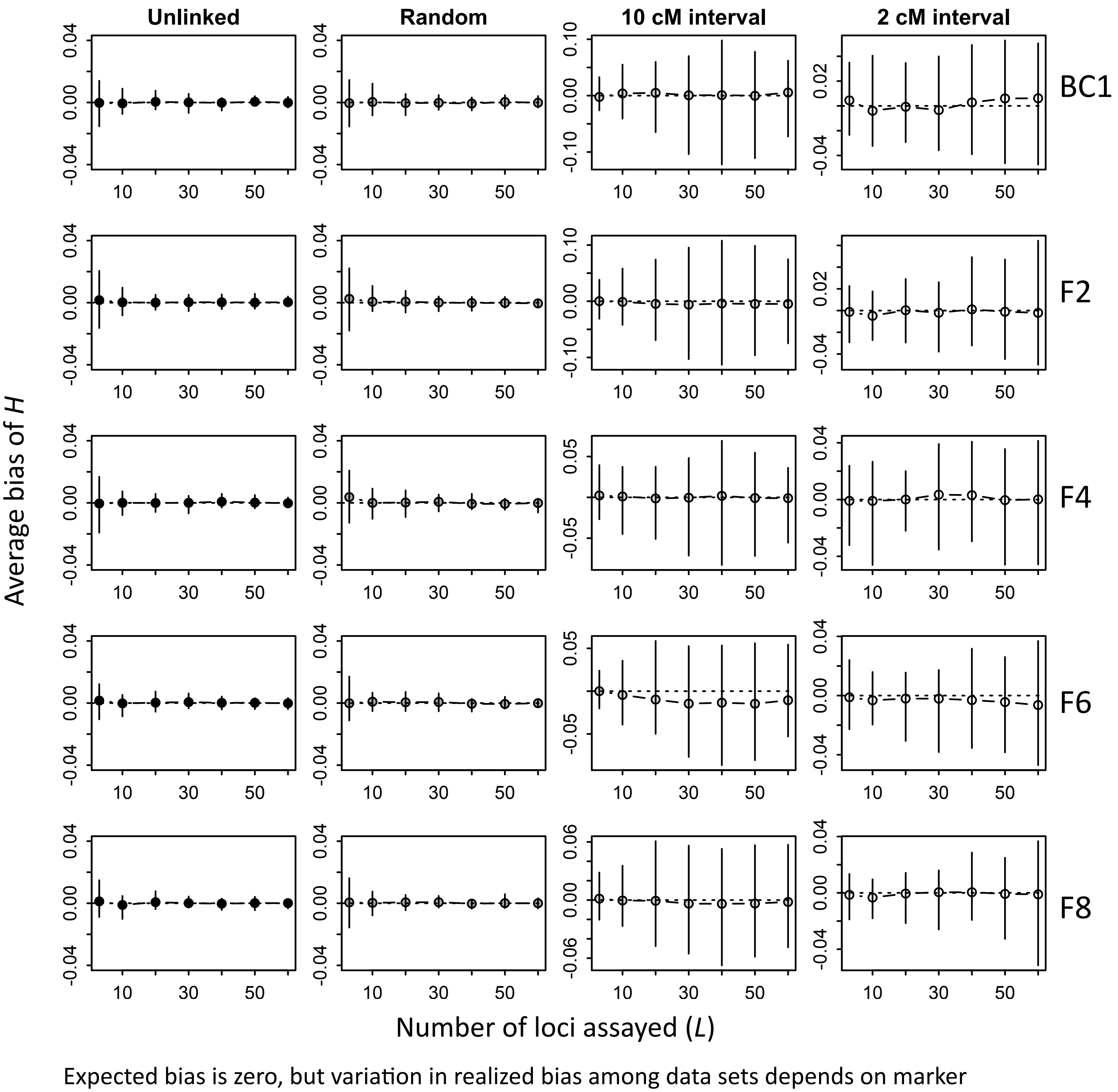

Expected bias is zero, but variation in realized bias among data sets depends on marker number and sampling regime. Means and 95% interquantile ranges (vertical bars) are illustrated for 1000 simulated individuals of each hybrid generation (right margin).

Figure S3 – Effect of linkage on sampling variance of ancestry estimates

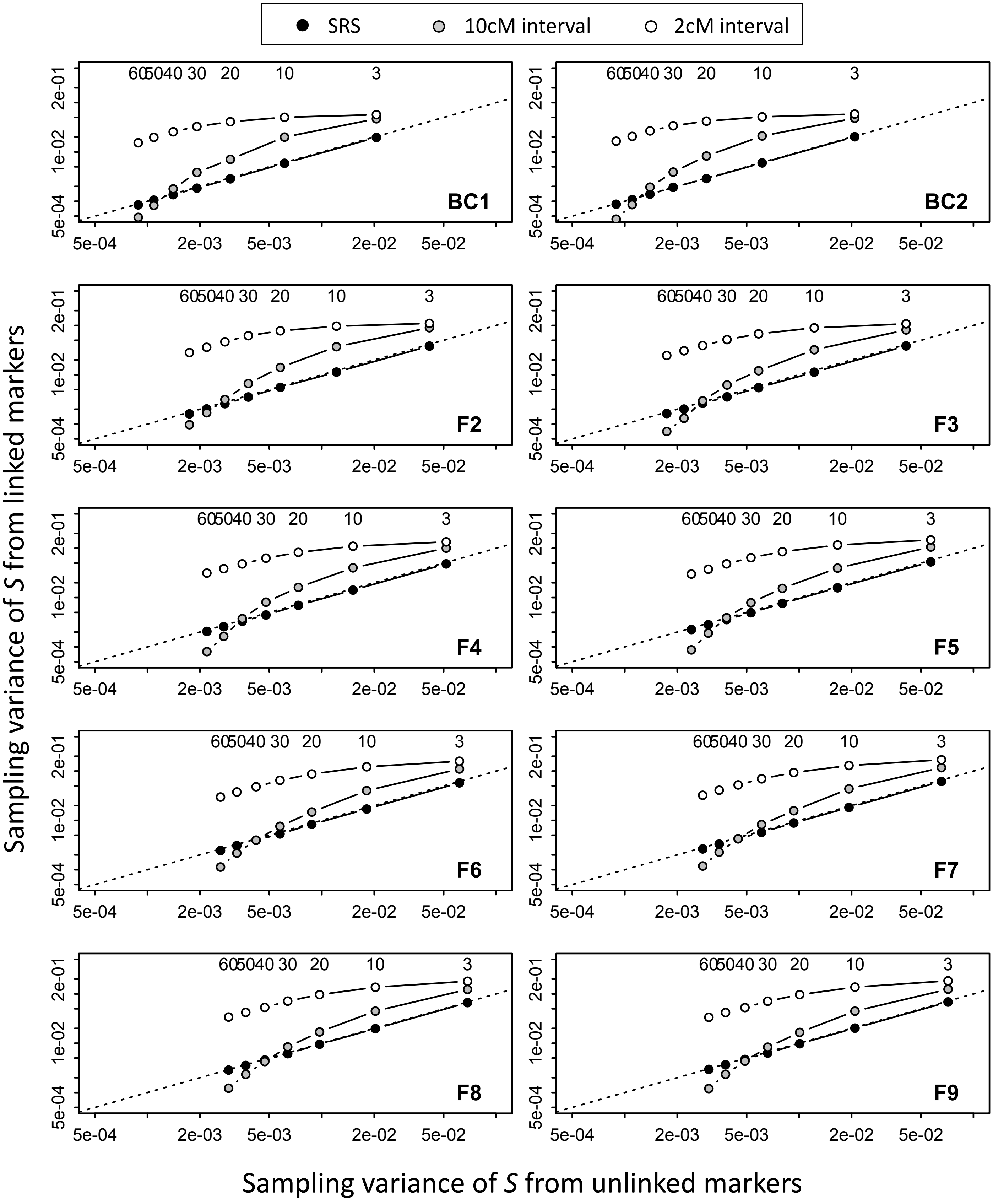

Figure S3 – Sampling variances of ancestry (S) estimates based on linked markers are plotted against the corresponding sampling variance of estimates based on unlinked markers (x-axis). SRS stands for simple random sampling of markers from the structured genome model. Dashed diagonal line represents equality of sampling variances. Numerals along the top of each panel indicate the number of markers sampled. Each panel displays averages from 1000 simulated individuals of the hybrid generation indicated in the lower right corner. Sampling variance for each individual for each sampling regime was estimated from 1000 independent samples of the individual's genome. These are the same data as Figures S1 and S2.

Figure S4 – Effect of linkage on sampling variance of heterozygosity estimates

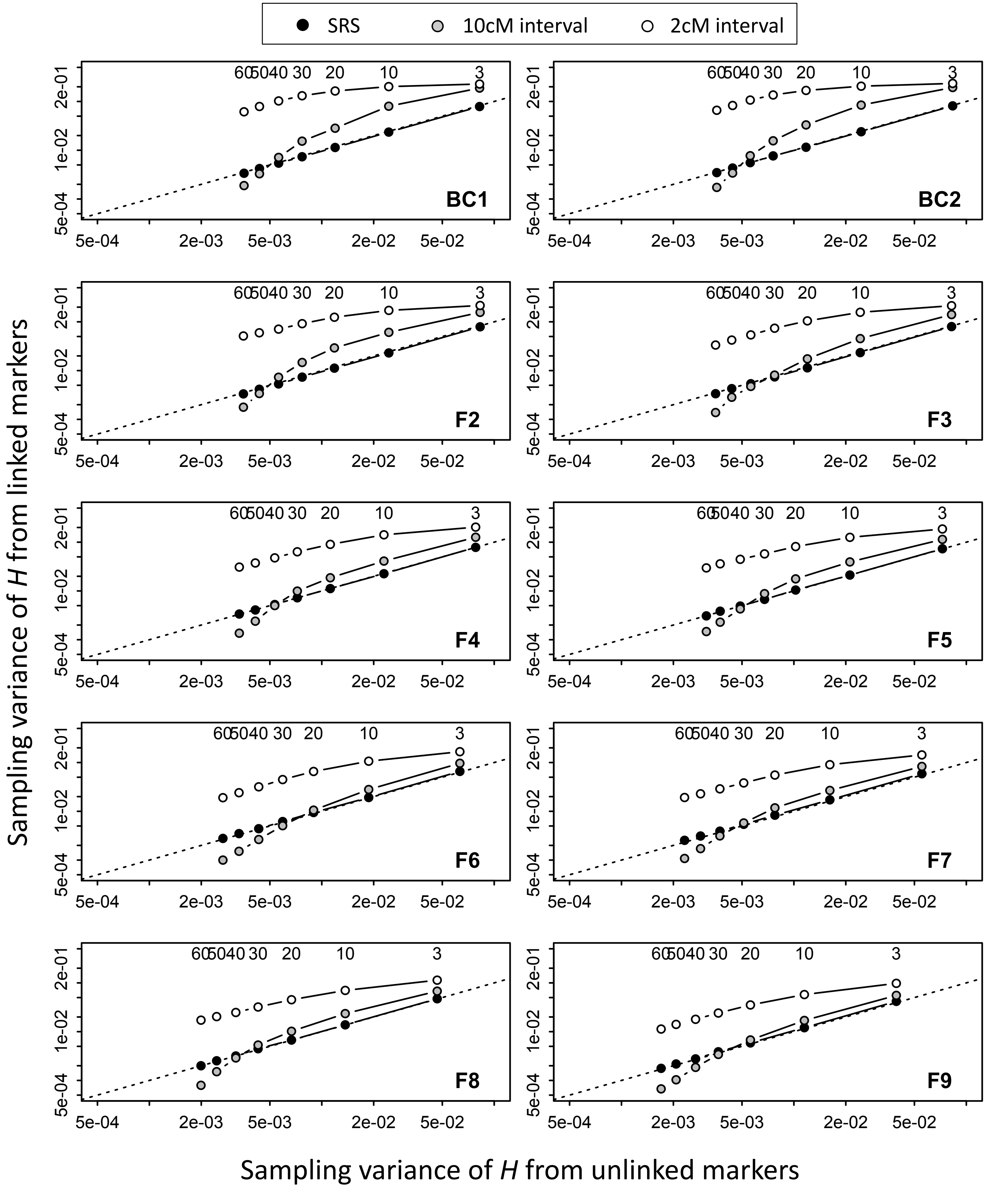

Figure S4 – Sampling variances of interclass heterozygosity (H_I) estimates based on linked markers are plotted against the corresponding sampling variance of estimates based on unlinked markers (x-axis) as in Figure S3. SRS stands for simple random sampling of markers from the structured genome model. Dashed diagonal line represents equality of sampling variances. Numerals along the top of each panel indicate the number of markers sampled. Each panel displays averages from 1000 simulated individuals of the hybrid generation indicated in the lower right corner. Sampling variance for each individual for each sampling regime was estimated from 1000 independent samples of the individual's genome. These are the same data as Figures S1 - S3.

Figure S5 – Effects of systematic error in assumed parental allele frequencies on ancestry estimates (3 markers)

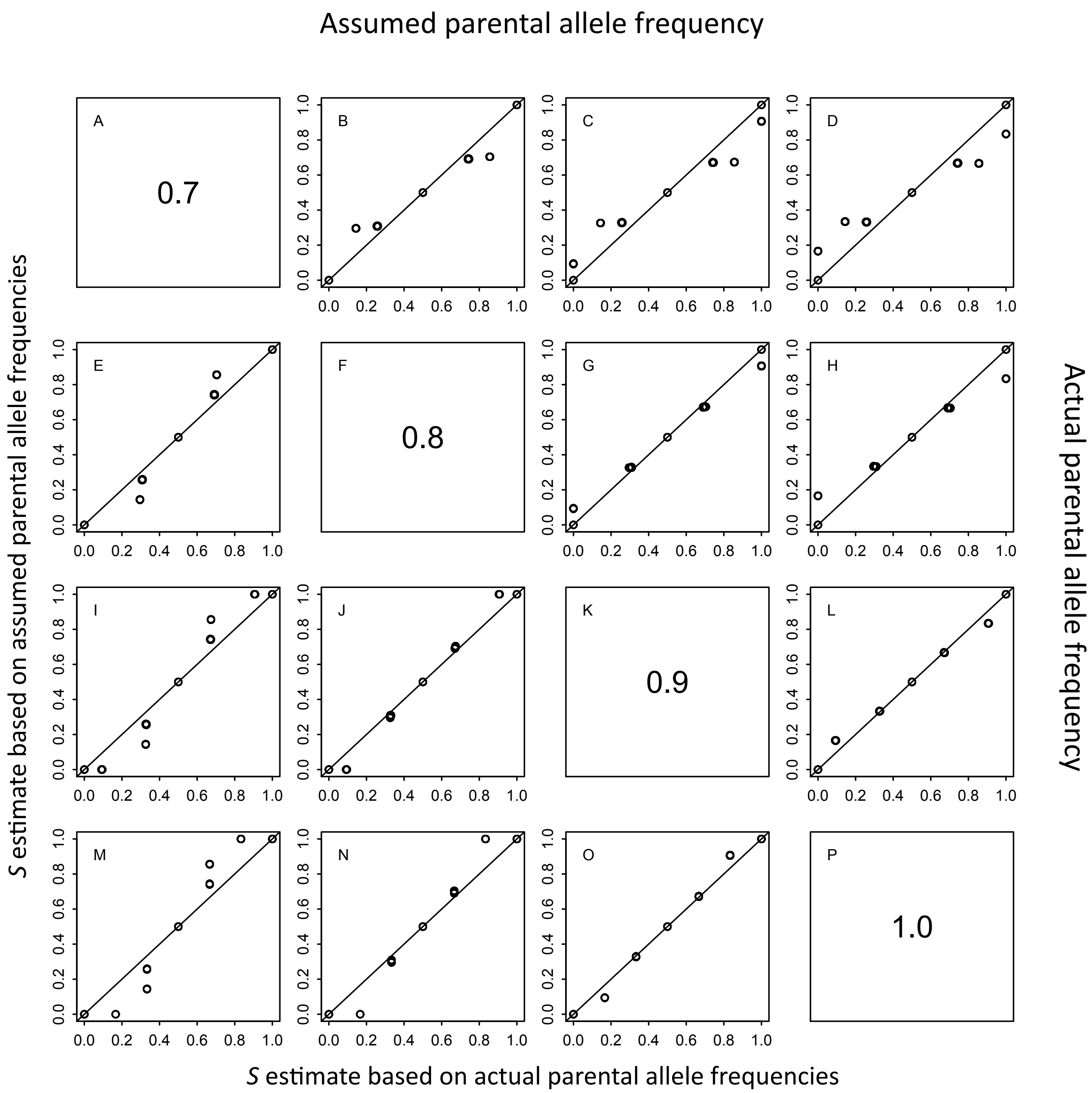

Figure S6 – Effects of systematic error in assumed parental allele frequencies on heterozygosity estimates (3 markers)

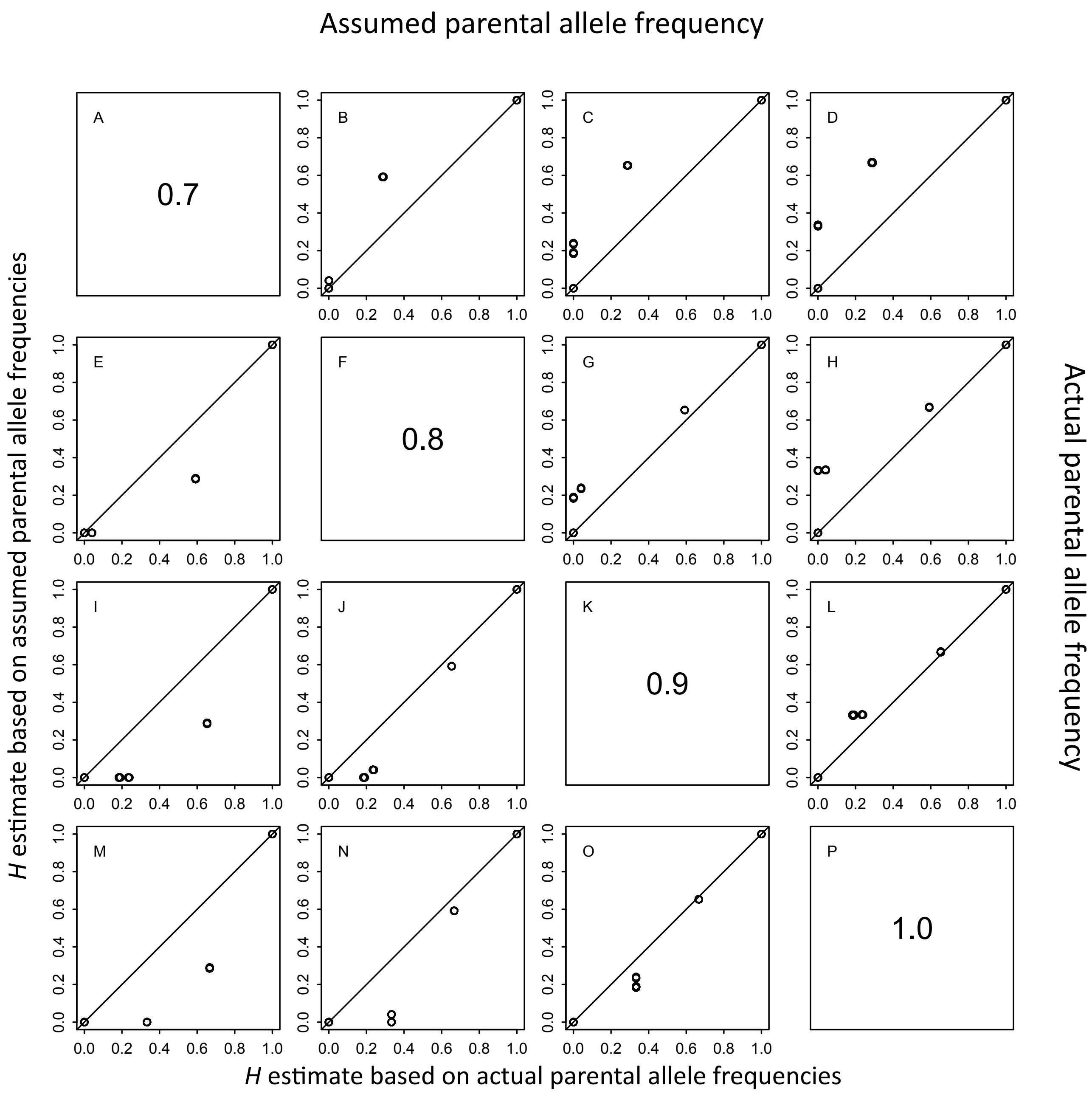

Figure S7 – Effects of systematic error in assumed parental allele frequencies on ancestry estimates (50 markers)

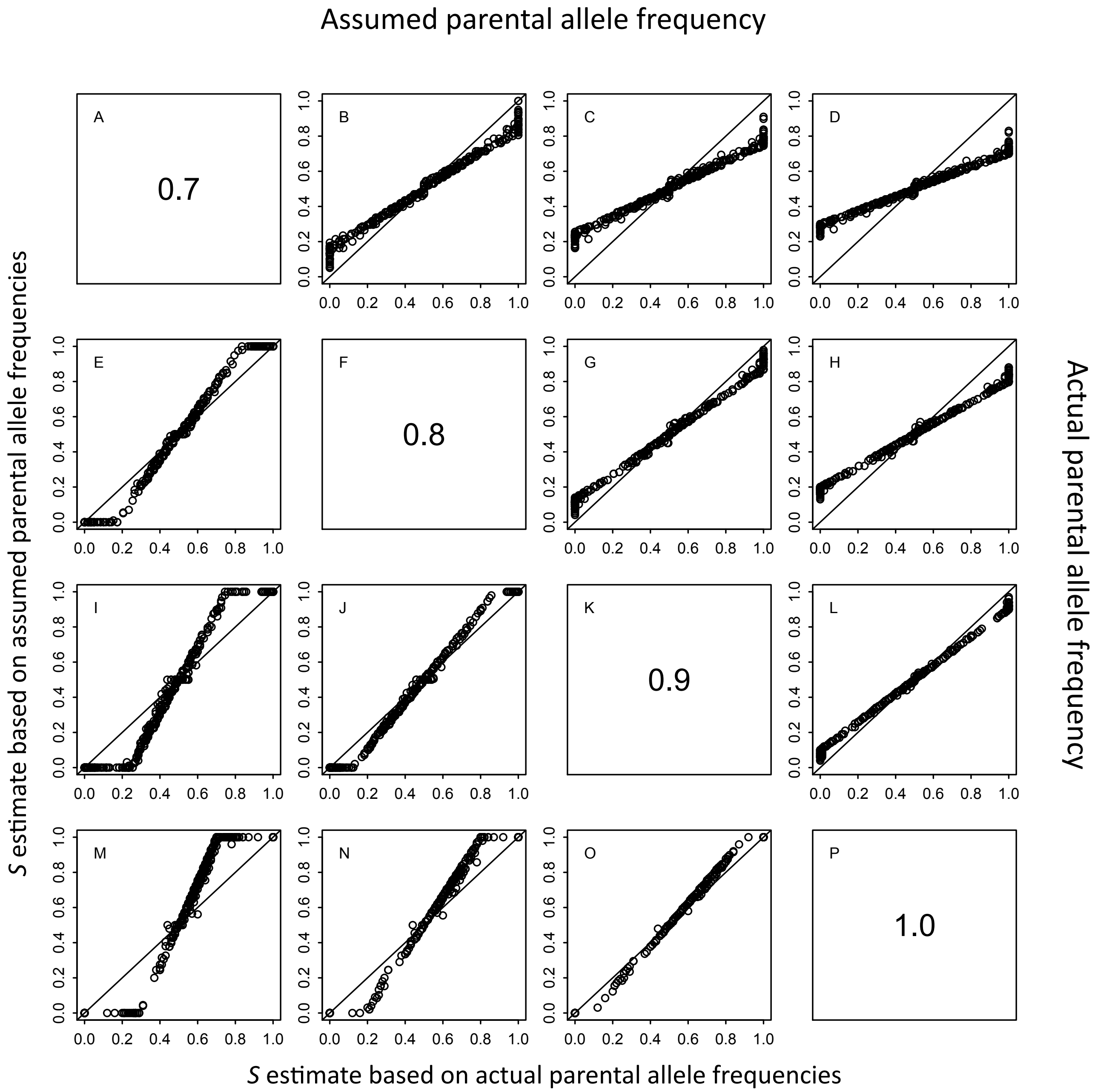

Figure S8 – Effects of systematic error in assumed parental allele frequencies on heterozygosity estimates (50 markers)

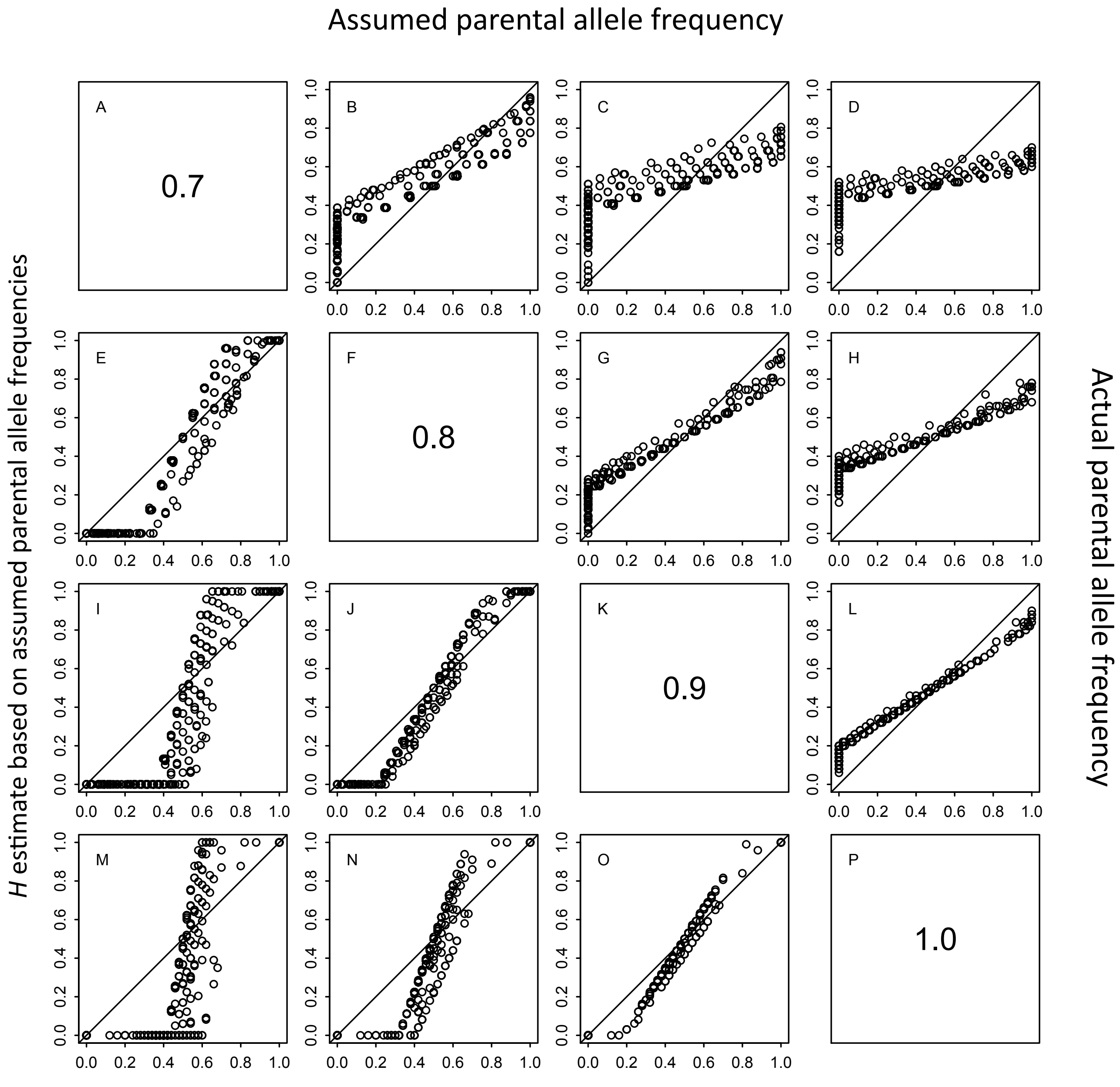

Figure S9 – Effects of balanced error in assumed parental allele frequencies on ancestry estimates (50 markers)

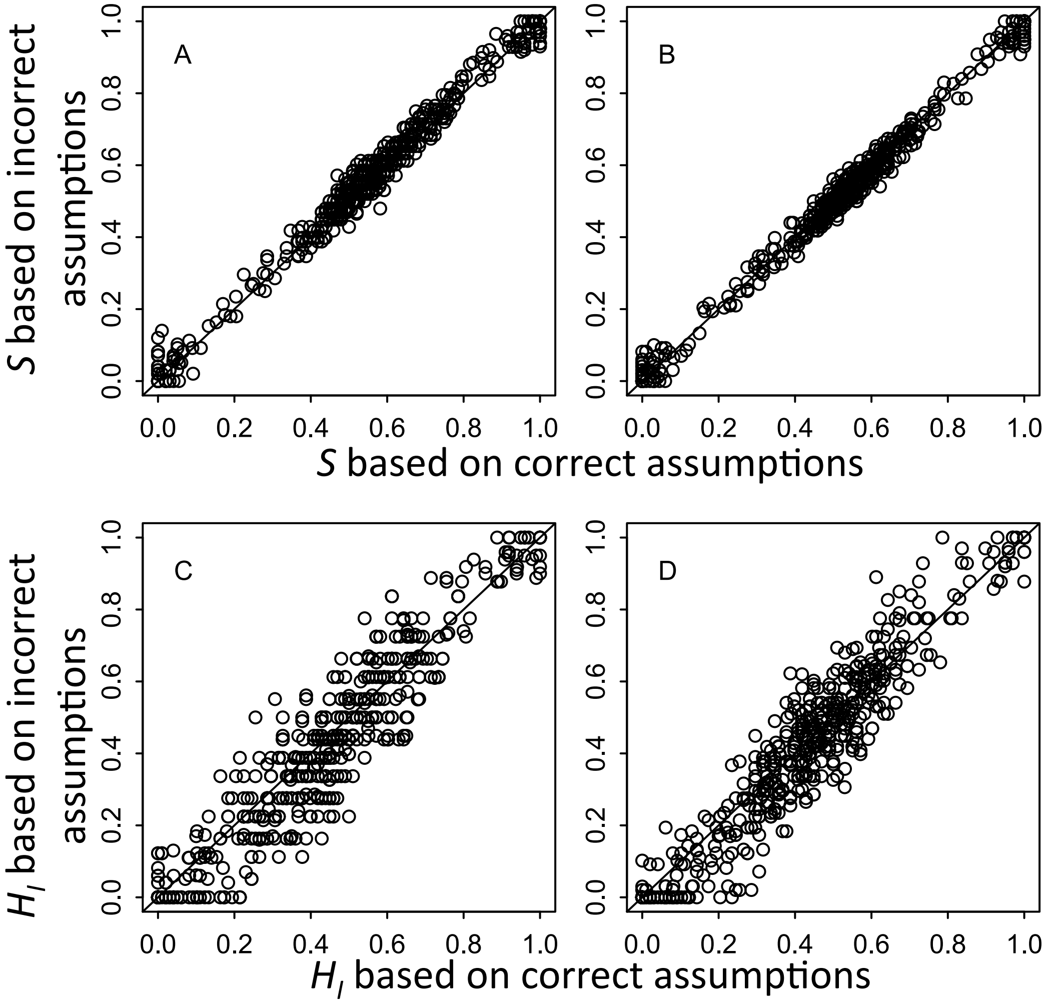

Table S1 - Effects of systematic error in assumed parental allele frequencies on ancestry estimates (3 markers)

For the actual and assumed parental frequencies given in the first two columns, estimates of S are compared to estimates based on the correct assumption using reduced major axis regression (RMA). Elevation and slope of the regression (with 95% confidence limits) were estimated from 600 simulated hybrid and parental individuals across 10 generations of admixture. Results are Illustrated in Figure S5.

Actual	Assumed	Elevation	lower	upper	Slope	lower	upper
0.70	0.70	0.00			1.00		
0.70	0.80	0.07	0.06	0.08	0.86	0.84	0.87
0.70	0.90	0.13	0.12	0.14	0.73	0.72	0.75
0.70	1.00	0.17	0.16	0.18	0.66	0.64	0.67
0.80	0.70	-0.06	-0.07	-0.05	1.13	1.11	1.15
0.80	0.80	0.00			1.00		
0.80	0.90	0.07	0.06	0.07	0.87	0.86	0.87
0.80	1.00	0.11	0.10	0.12	0.78	0.77	0.79
0.90	0.70	-0.11	-0.13	-0.10	1.22	1.19	1.24
0.90	0.80	-0.07	-0.07	-0.06	1.12	1.11	1.13
0.90	0.90	0.00			1.00		
0.90	1.00	0.04	0.04	0.05	0.92	0.92	0.93
1.00	0.70	-0.07	-0.09	-0.05	1.20	1.18	1.23
1.00	0.80	-0.04	-0.06	-0.03	1.12	1.11	1.14
1.00	0.90	-0.02	-0.02	-0.01	1.05	1.04	1.05
1.00	1.00	0.00			1.00		

Table S2 - Effects of systematic error in assumed parental allele frequencies on heterozygosity estimates (3 markers)

For the actual and assumed parental frequencies given in the first two columns, estimates of H are compared to estimates based on the correct assumption using RMA as in Table S1. Results are Illustrated in Figure S6.

Actual	Assumed	Elevation	lower	upper	Slope	lower	upper
0.70	0.70	0.00			1.00		
0.70	0.80	0.08	0.06	0.09	1.20	1.16	1.24
0.70	0.90	0.20	0.18	0.21	1.07	1.03	1.11
0.70	1.00	0.27	0.25	0.28	0.99	0.95	1.03
0.80	0.70	-0.06	-0.07	-0.05	0.86	0.83	0.88
0.80	0.80	0.00			1.00		
0.80	0.90	0.13	0.12	0.13	0.89	0.88	0.91
0.80	1.00	0.20	0.19	0.21	0.83	0.81	0.86
0.90	0.70	-0.15	-0.17	-0.14	0.95	0.92	0.99
0.90	0.80	-0.12	-0.13	-0.11	1.09	1.07	1.11
0.90	0.90	0.00			1.00		
0.90	1.00	0.07	0.07	0.08	0.95	0.94	0.97
1.00	0.70	-0.18	-0.20	-0.16	0.98	0.94	1.02
1.00	0.80	-0.14	-0.16	-0.13	1.08	1.05	1.12
1.00	0.90	-0.05	-0.06	-0.04	1.03	1.01	1.04
1.00	1.00	0.00			1.00		

Table S3 - Effects of systematic error in assumed parental allele frequencies on ancestry estimates (50 markers)

For the actual and assumed parental frequencies given in the first two columns, estimates of S are compared to estimates based on the correct assumption using RMA as in Table S1. Results are Illustrated in Figure S7.

Actual	Assumed	Elevation	lower	upper	Slope	lower	upper
0.70	0.70	0.00			1.00		
0.70	0.80	0.14	0.14	0.14	0.72	0.71	0.73
0.70	0.90	0.23	0.22	0.23	0.55	0.54	0.55
0.70	1.00	0.28	0.28	0.28	0.44	0.44	0.45
0.80	0.70	-0.06	-0.07	-0.05	1.12	1.11	1.13
0.80	0.80	0.00			1.00		
0.80	0.90	0.11	0.10	0.11	0.79	0.78	0.79
0.80	1.00	0.18	0.18	0.18	0.64	0.63	0.64
0.90	0.70	-0.13	-0.15	-0.12	1.22	1.19	1.24
0.90	0.80	-0.05	-0.06	-0.05	1.09	1.08	1.10
0.90	0.90	0.00			1.00		
0.90	1.00	0.09	0.09	0.09	0.83	0.82	0.83
1.00	0.70	-0.05	-0.07	-0.03	1.23	1.19	1.26
1.00	0.80	-0.02	-0.04	-0.01	1.11	1.09	1.13
1.00	0.90	-0.01	-0.01	-0.00	1.04	1.03	1.04
1.00	1.00	0.00			1.00		

Table S4 - Effects of systematic error in assumed parental allele frequencies on heterozygosity estimates (50 markers)

For the actual and assumed parental frequencies given in the first two columns, estimates of H are compared to estimates based on the correct assumption using RMA as in Table S1. Results are Illustrated in Figure S8.

Actual	Assumed	Elevation	lower	upper	Slope	lower	upper
0.70	0.70	0.00			1.00		
0.70	0.80	0.19	0.18	0.20	0.68	0.66	0.70
0.70	0.90	0.32	0.31	0.33	0.41	0.40	0.43
0.70	1.00	0.38	0.38	0.39	0.27	0.26	0.28
0.80	0.70	-0.16	-0.17	-0.14	1.27	1.23	1.30
0.80	0.80	0.00			1.00		
0.80	0.90	0.19	0.18	0.19	0.65	0.64	0.66
0.80	1.00	0.30	0.30	0.31	0.41	0.40	0.42
0.90	0.70	-0.24	-0.27	-0.21	1.36	1.30	1.42
0.90	0.80	-0.10	-0.11	-0.09	1.17	1.15	1.20
0.90	0.90	0.00			1.00		
0.90	1.00	0.16	0.16	0.17	0.68	0.67	0.69
1.00	0.70	-0.26	-0.29	-0.22	1.30	1.23	1.37
1.00	0.80	-0.12	-0.14	-0.10	1.16	1.12	1.20
1.00	0.90	-0.03	-0.04	-0.03	1.04	1.03	1.06
1.00	1.00	0.00			1.00		

Table S5 - Effects of balanced error in assumed parental allele frequencies (50 markers)

For assumed parental frequencies of 0.8 and 0.2, estimates of S and H_I are compared to estimates based on the correct assumption using RMA as in Table S1. In scenario 1, 25 of the markers actually had parental frequencies of 0.9 and 0.1 while the other 25 markers had actual frequences of 0.7 and 0.3. In scenario 2, one marker of each frequency class was replaced by a truly diagnostic marker. Results are Illustrated in Figure S9.

scenario	index	Elevation	lower	upper	Slope	lower	upper
1	S	0.01	0.01	0.02	1.00	0.99	1.01
2	S	-0.00	-0.01	0.00	0.99	0.98	1.00
1	H	-0.04	-0.05	-0.03	1.06	1.03	1.08
2	H	-0.04	-0.05	-0.03	1.07	1.04	1.09
